# New Nanosized Systems Doxorubicin—Amphiphilic Copolymers of *N*-Vinylpyrrolidone and (Di)methacrylates with Antitumor Activity

**DOI:** 10.3390/pharmaceutics14122572

**Published:** 2022-11-23

**Authors:** Svetlana V. Kurmaz, Vladislav M. Ignatiev, Nina S. Emel’yanova, Vladimir A. Kurmaz, Dmitry V. Konev, Anastasiya A. Balakina, Alexey A. Terentyev

**Affiliations:** 1 Federal Research Center of Problems of Chemical Physics and Medicinal Chemistry, Russian Academy of Sciences, ac. Semenov av. 1, 142432 Chernogolovka, Russia; 2 Faculty of Fundamental Physical and Chemical Engineering, Moscow State University M.V. Lomonosov, GSP-1, Leninskie Gory, 1, Building 51, 1 Educational Building, 119991 Moscow, Russia; 3 Scientific and Educational Center of the Moscow State Regional University, St. Vera Voloshina 24, 141014 Mytishchi, Russia

**Keywords:** *N*-vinylpyrrolidone, (di)methacrylates, amphiphilic copolymers, doxorubicin, quantum chemical modeling, hydrogen bonding, cyclic voltammetry, cytotoxicity, cell accumulation, drug delivery

## Abstract

Nanosized systems of DOX with antitumor activity on the base of micelle-like particles of amphiphilic thermosensitive copolymers of *N*-vinylpyrrolidone (VP) with triethylene glycol dimethacrylate (TEGDM), and *N*-vinylpyrrolidone and methacrylic acid (MAA) with TEGDM were explored. They were investigated in aqueous solutions by electron absorption spectroscopy, dynamic light scattering and cyclic voltammetry. Experimental data and quantum-chemical modeling indicated the formation of a hydrogen bond between oxygen-containing groups of monomer units of the copolymers and H-atoms of OH and NH_2_ groups of DOX; the energies and H-bond lengths in the considered structures were calculated. A simulation of TDDFT spectra of DOX and its complexes with the VP and TEGDM units was carried out. Electrochemical studies in PBS have demonstrated that the oxidation of encapsulated DOX appeared to be easier than that of the free one, and its reduction was somewhat more difficult. The cytotoxicity of VP-TEGDM copolymer compositions containing 1, 5 and 15 wt% DOX was studied in vitro on *HeLa* cells, and the values of IC_50_ doses were determined at 24 and 72 h of exposure. The copolymer compositions containing 5 and 15 wt% DOX accumulated actively in cell nuclei and did not cause visual changes in cell morphology.

## 1. Introduction

An important goal of practical medicine is the targeting delivery of efficient but insufficiently selective drugs into damaged cells. Doxorubicin (DOX) that is widely used to prevent a broad range of tumors belongs to them as well. However, its high general toxicity and high allergenicity together with considerable cardiotoxicity restrict a therapeutic use of this drug and determine a necessity of its targeting delivery just into damaged cells. Quite a broad range of inorganic and organic materials are considered as carriers of drugs, in part, DOX and its derivatives. They are nanotubes [1], apoferritin [2], mesoporous silica nanoparticles [3], nanoparticles of complex composition based on SiO_2_ [4], vaterite (CaCO_3_) [5,6] including dextrane sulphate and poly-L-arginine [6], natural biopolymers (chitosan [7]), synthetic biocompatible polymers and copolymers [8,9], nanoparticles of polypyrrole and poly(2-diethylamino)ethyl methacrylate [10], dextrin nanogels [11], lipoic acid derivative of cyclodextrin [12,13], etc. More details can be found in the reviews [14,15].

Poly-*N*-vinylpyrrolidone (PVP) (commercial name is polyvidone/povidone) is among the most popular carriers. It has unique physical and chemical properties: it is chemically inert, colorless, heat-resistant and pH-stable; has excellent solubility in water and organic solvents of different polarity; and good sorption properties [16]. It is recognized by the Food and Drug Administration (FDA) as a safe polymer, and therefore it is widely used in medicine, cosmetics, pharmaceutics, etc. [17,18,19,20,21]. PVP is used to develop various drug delivery systems intended for oral, local, transdermal and intravenous administration [22]; is of interest for gene delivery [23,24,25,26,27]; targeted delivery of biologically active compounds (BAC) of various nature [28,29]; and can be used in regenerative medicine [30,31,32]. The polymer provides controlled release of the drug, improves the bioavailability of poorly water-soluble drugs and protects the active compound from external factors (pH, temperature and oxygen). These valuable properties can also be retained in VP-based copolymers with a complex architecture of macromolecules, and new properties may appear due to their topology. Currently, the importance of amphiphilic polymers of complex architecture (dendrimers [33], hyperbranched, star-shaped, etc. [34,35,36]) as BAC carriers and delivery vehicles is increasing. Due to their topology, they provide a prolonged action of drugs, change their distribution in the body, diminish toxicity, etc.

Cross-linking radical copolymerization of mono- and polyfunctional monomers within the conditions of the restriction of primary polymer chains with the aid of conventional (thiols of different types) and catalytic chain transfer agents is one of the most effective, one-step methods to prepare branched copolymers [37,38,39,40,41]. It was used to synthesize branched VP copolymers without terminal S-containing groups [42], which is very important for obtaining biocompatible copolymers. Amphiphilic copolymers of *N*-vinylpyrrolidone with branching inside chains and controlled molecular weight, amphiphilicity, macromolecule size and self-organizing ability are of interest for medicine, pharmaceutics and cosmetics. Unlike traditional PVP, they are characterized by: (i) amphiphilicity, which is easy to control due to the monomer composition and the comonomer nature; (ii) the ability of copolymers of some composition to respond to external conditions (temperature and pH); (iii) the ability of hydrolysis in acidic and alkaline media along C-O bonds of dimethacrylate units; (iv) sufficiently small size of individual macromolecules and their aggregates in water will facilitate their efficient cellular and tissue uptake. Nanosized systems of metformin as a well-known antidiabetic agent [43], lipophilic organic complexes of platinum(IV) with antitumor activity [44,45] and zinc tetraphenylporphyrinate as a means of intracellular accumulation visualizing [42] were obtained on their basis. The high biocompatibility of polymer carriers and their ability to penetrate into *HeLa* cells have been demonstrated in vitro, which indicates that the developed delivery materials are promising for the intracellular delivery of BAC. During in vivo experiments, the VP-TEGDM copolymer does not exhibit any toxicity, as well as its complex with metformin [46].

**Figure 1:**
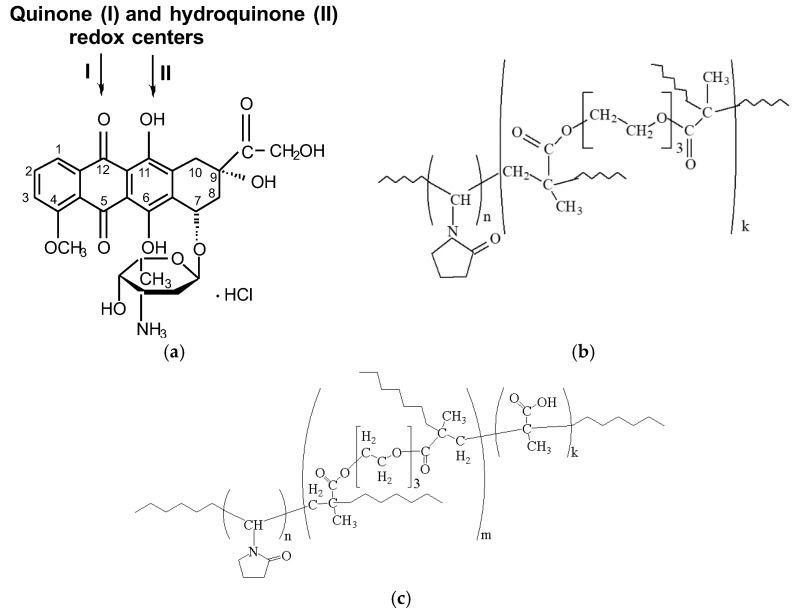
Chemical formulas of DOX (**a**), VP-TEGDM (**b**) and VP-MAA-TEGDM copolymers (**c**).

Anthracycline antibiotic DOX (pK_a_ 8.6) ((8*S*,10*S*)-10-(4-amino-5-hydroxy-6-methyl-tetrahydro-2*H*-pyran-2-yloxy)-6,8,11-trihydroxy-8-(2-hydroxyacetyl)-1-methoxy-7,8,9,10-tetrahydrotetracene-5,12-dione), also called adriamycin, is a very efficient anticancer agent with a wide range of applications [47]. The high chemical activity of DOX is determined mainly by two reactive centers in the molecule, namely, quinone and hydroquinone (Figure 1a). DOX is a polar molecule with high charge delocalization, and it is a good acceptor of electrons [48].

DOX has antimitotic and antiproliferative effects. The antitumor effect is due to its inclusion into the double helix of DNA and the anthracycline moiety in cancer cells and these, consequently, produce the inhibition of the replication and transcription of DNA, an appearance of one- and two-chains breaking in DNA, a formation of free radicals and direct action on cell membranes to suppress a synthesis of nucleic acids [15]. However, the exact mechanism of its action is complex and still not quite clear. It shows high efficiency in relation to many types of oncological diseases, but it is able to accumulate in both damaged and healthy cells and exhibits high cardiotoxicity. In this regard, many studies are devoted to the development of approaches to increasing its selectivity and circulation time in the bloodstream [15]. In recent years, the DOX incorporation into liposomes, micelles and hydrogels; micro- and nanoencapsulation into chitosan and its derivatives; its complexation with polyacrylic, polyaspartic and polyglutamic acids, etc.; as well as covalent and non-covalent immobilization in polymers have been actively studied [47,49,50,51].

The aim of this work is to prepare and characterize via different physical chemical methods new nanosized DOX systems based on amphiphilic branched copolymers of *N*-vinylpyrrolidone with triethylene glycol dimethacrylate (VP-TEGDM), and VP-MAA-TEGDM where MAA is methacrylic acid, and to perform quantum-chemical modeling of the structure of DOX–copolymer complexes and study the biological activity of prepared structures in vitro on *HeLa* tumor cells.

## 2. Materials and Methods

### 2.1. Materials

We used a VP-TEGDM copolymer (CPL1) obtained by radical copolymerization in toluene at a monomer molar ratio of 100:5 as described in [25], and a VP-MAA-TEGDM copolymer (CPL2) synthesized from a 98:2:2 monomer mixture in ethanol [52]. The copolymers were isolated from the reaction mixture by precipitation with a 10-fold excess of hexane. The obtained copolymers were described in detail elsewhere [42,52].

The monomer VP (Alfa Aesar, Haverhill, MA, USA) was purified by vacuum distillation to remove the NaOH inhibitor. The monomers TEGDM and MAA (Aldrich, St. Louis, MO, USA), isopropyl alcohol (IPA, Khimmed, Moscow, Russia) of the extra purity grade as well as Na_2_HPO_4_ and NaH_2_PO_4_ were used without additional purification. The initiator, azobisisobutyronitrile (AIBN), was purified by recrystallization from ethanol.

All studies were carried out with the commercial drug doxorubicin hydrochloride (Doxorubicin-Teva, Pharmachemie B.V., Haarlem, The Netherlands, with lactose monohydrate as an excipient), which was used as received. Double deionized water, neutral phosphate buffer solutions and PBS with pH 6.8–7.0 (for DLS experiments) or 7.24 (for CVA experiments), were used to prepare DOX–copolymer composition solutions for all physicochemical experiments. In biology studies (cellular accumulation of nanoparticles in cell), PBS with pH 7.4 (137 mM NaCl, 2.68 mM KCl, 4.29 mM Na_2_HPO_4_, 1.47 mM KH_2_PO_4_,) was used as received.

### 2.2. The Methods of the Formation of DOX–Copolymer Compositions

The DOX–copolymer compositions based on the VP-TEGDM copolymer were produced by two methods. In *the first manner*, an aqueous DOX solution was added dropwise to the copolymer solution in isopropyl alcohol (IPA) with constant stirring using a magnetic stirrer. The DOX content was varied from 0.4 to 15 wt% per the copolymer. The concentrations of the reagents and the DOX encapsulation conditions are given in Table S1. After evaporation of the solvents in air and in vacuo, dry copolymer films with DOX were dissolved in PBS with pH 6.8 or 7.0, and the resulting solutions were analyzed. In the control experiment (run #3), the copolymer was absent in the solution; the DOX concentration corresponded to the concentration of the solution obtained in the presence of the copolymer (run #1). The samples obtained in runs #2, 7 and 8 were used for biological tests.

In *the second manner*, the VP-TEGDM copolymer was solved in double deionized water (1 mg mL^−1^); the solution was diluted up to the concentrations give in Table S2. Then, 0.1 mL of a DOX solution in water was added dropwise to the copolymer solutions. As a result, the DOX content per the copolymer varied over a wide range, from 2.5 to 40 wt%. After drying, copolymer films with DOX were dissolved in PBS with pH 6.8.

To prepare a DOX composition based on the VP–MAA-TEGDM copolymer (CPL2), 2.75 mL of the DOX solution was added dropwise to 2 mg mL^−1^ of the solution in 44 mL of IPA with constant stirring using a magnetic stirrer. The DOX content was 3.1 wt% per the copolymer. The composition was dried from solvents in air and vacuum until a constant weight. The resulting polymer composition was used for electrochemical measurements.

### 2.3. The Methods

#### 2.3.1. Elemental Analysis

The content of CHNS atoms in copolymers was determined by elemental analysis on a CHNS/O instrument Vario MICRO cube (Elementar Analysensysteme GmbH, Langenselbold, Germany, 2007). The nitrogen content in VP-TEGDM and VP-MAA-TEGDM copolymers is 9.94 and 9.83%.

#### 2.3.2. Potentiometry

Potentiometric titration of the aqueous solution of the VP-MAA-TEGDM copolymer was performed using Multitest IPL-201 (SEMIKO, Novosibirsk, Russia) ionomer (titrant was 0.01 M NaOH solution).

#### 2.3.3. IR- and ^1^H NMR Spectroscopy

The copolymers were identified using IR- and ^1^H NMR-spectroscopy. The IR spectra of the copolymers were recorded on a Bruker α FTIR instrument in the transmission mode. The ^1^H NMR spectra of the copolymers in deuterated chloroform (6 mg mL^−1^) were recorded on an AVANCE III 500 MHz BRUKER BioSpin superconducting pulsed broadband two-channel NMR spectrometer (Billerica, MA, USA) using glass ampoules that were 5 mm in diameter.

#### 2.3.4. Electronic Absorption Spectroscopy

The absorption spectra of aqueous solutions of DOX, the copolymer and DOX-copolymer compositions were recorded in UV and visible regions on a SPEKS instrument (SPEKS SSP-705-1, “Spectroscopic Systems”, Moscow, Russia) using cuvettes of 0.2, 0.5 or 1 cm thickness.

#### 2.3.5. Size Exclusion Chromatography

The absolute molecular weights of the copolymers were determined by size exclusion chromatography using a Waters liquid chromatograph (2 columns PS-gel, 5 μm, MIXED-C, 300 × 7.5 mm) (Waters Corp, Milford, MA, USA) equipped with a refractive index detector and a multi-angle light scattering detector WYATT DAWN HELEOS II, (Wyatt Technology, Santa Barbara, CA, USA), λ = 658 nm. The eluent was *N*-methylpyrrolidone with the addition of lithium chloride (1 wt%), which prevents the aggregation of macromolecules in a polar solvent. The measurement temperature was 70 °C, and the elution rate was 1 mL min^−1^. The d*n*/d*c* values were determined from multi-angle light scattering detector data. All copolymer solutions (10–20 mg mL^−1^) were preliminarily filtered through filters with a pore diameter of 0.2 μm. The absolute weight average molecular weight of the copolymer was obtained from light scattering data using Astra software version 5.3.2.20 (Wyatt Technology).

#### 2.3.6. Dynamic Light Scattering

The hydrodynamic radii, *R*_h_, of copolymers and DOX–copolymer structures in IPA, water and PBS were determined by dynamic light scattering (DLS). Before preparing the samples for measurements, the solutions were filtered using a filter with a pore diameter of 0.45 μm, and the vials with the solution were thermostated at a given temperature for 20 min. A temperature range for measurements was 20–50 °C. DLS measurements were carried out using a Photocor Compact instrument (Photocor LTD, Moscow, Russia) equipped with a diode laser operating at a wavelength of 654 nm. The solutions of copolymers and DOX–copolymer structures were analyzed at a detection angle of 90 °C. The experimental data were processed using the DynaLS software, version 2.8.3 (Dr. Alexander A Goldin (Alango Ltd., Hefa, Israel). Updated in March 2002). The size distribution curves for scattering centers were obtained by processing the results of measurements of scattering intensity fluctuations by solutions. The hydrodynamic radii *R*_h_ of the copolymers were calculated using the Einstein–Stokes equation

*D* = *kT*/6πη*R*

where *D* is the diffusion coefficient, *k* is the Boltzmann constant, *T* in the equation is the absolute temperature (K) and η is the viscosity of the medium, in which the dispersed particles are suspended.

#### 2.3.7. Electrochemical Measurements

The electrochemical experiments with an Autolab/PGSTAT302N universal high-speed potentiostat/galvanostat (ECOCHEMIE, Utrecht, The Netherlands) were carried out by cyclic voltammetry (CVA) in a three-electrode undivided electrochemical glass cell; the working volume was from 5 to 15 cm^3^. Before the experiments, the working solutions were deaerated by alternately applying a vacuum and filling the electrochemical cells with argon. When the components of a working solution and electrodes were introduced into the cell, as well as during the electrochemical measurements, a slight excess argon pressure (~20 mbar) was maintained above the solutions. The standard Schlenk procedure [44,53,54,55] was used for it. All the experiments were performed at a room temperature in PBS with pH 7.24, and the range of the potential scan rate was 10–2000 mV∙s^–1^. The working electrode was an HTW Segradur-G (HTW Hochtemperatur-Werkstoffe GmbH, Thierhaupten, Germany) glassy carbon (GC) disc electrode with a diameter of 3 mm soldered into a glass. The auxiliary electrode was a Pt wire, and the reference electrode was a silver/silver chloride electrode (Ag/AgCl/KCl_sat._), and all the potentials in this work were referred with it. The GC electrode was polished immediately before an experiment by a diamond suspension (diameter of 1 μm and after of 0.25 μm), then rinsed with ultrasonic machining. Because DOX adsorbs on a GC strongly and irreversibly, in accordance with recommendation [56], it is always necessary to polish and clean the electrode surface very thoroughly. Therefore, we added one more stage into the electrode preparation before experiments, namely, its cleaning with H_2_SO_4_/H_2_O_2_ admixture followed by an ethyl alcohol washing. A detailed description of the CVA experiments can be found elsewhere [44,53,57].

### 2.4. Quantum-Chemical Modeling of DOX–Copolymer Complexes

Quantum-chemical calculations were carried out in several stages at different levels of the study of the electronic structure. At the first stage, using the semi-empirical AM1 method in the Gaussian 09 program [58] and in the DFT framework of the density functional theory (PBE/SBK) in the PRIRODA program [59], a copolymer section was modeled, consisting of the maximum amount of VP and TEGDM units available for calculations in the experimentally found molar ratio. The optimization of the DOX molecule was carried out in the Gaussian 09 program [58]. The hybrid functional TPSSh [60] and the basis set 6-31G*//6-311++G** were used as a method and basis. The modeling was carried out by the same methods as the sections of the copolymer. At the next stage, Gaussian 09 (TPSSh/311++G**//6-31G*) was used again to study the individual bonds formed using QTAIM and to model the TDDFT spectra. There were no imaginary vibration frequencies in the calculation results, and all optimized structures corresponded to the minimum potential energy. The influence of the solvent (water) was also taken into account using the polarizable continuum model (PCM).

To analyze the wave functions using the QTAIM method, the AIMALL software package (version 10.05.04) [61] was used. The wave functions of the structures were calculated in the same approximations as the geometry optimization of small sections of the copolymers. In particular, from the analysis of the wave functions, we found the energies of intermolecular bonds (*E*_bonds_), the electron density (ρ) and the Laplacian of the electron density (∇^2^ρ) at the critical points of the bond. The energies of intermolecular bonds were calculated using the formula *E*_a-b_ ≈ 1/2ν_e_(*r*) [62], where *E*_a-b_ is the A-B bond energy, and ν_e_(*r*) is the potential energy density at the critical point of the A-B bond. The theoretical UV and visible spectra were plotted using the TDDFT procedure (tpssh/6-31G*). The illustrations were made using the ChemCraft program (version 1.8, Grigoriy A. Andrienko, Southern Federal University, Rostov-on-Don, Russia) [63].

### 2.5. Biologic Study of DOX–Copolymer Compositions In Vitro

**Mammalian cell cultures.** Biological studies were performed on a *HeLa* cell culture (human cervical adenocarcinoma, *M-HeLa* clone) obtained from the collection of the Institute of Cytology, Russian Academy of Sciences.

**Cells cultivation.** Cell cultivation was carried out according to the standard method in an atmosphere of 5% CO_2_ and a temperature of 37 °C in an EMEM medium (PanEco, Moscow, Russia) with the addition of 10% fetal calf serum.

**Determination of cytotoxicity.** The study of cytotoxic properties was carried out using the MTT test. The studied cells were seeded into 96-well culture plates at a concentration of 5 × 10^4^ cells∙mL^−1^. Compounds were introduced into the culture medium 24 h after seeding. MTT dye (3-(4,5-dimethylthiazol-2-yl)-2,5-diphenyl-2H-tetrazolium bromide) was added to the incubation medium 24 or 72 h after the introduction of the test compounds at a concentration of 0.5 mg mL^−1^. The formazan crystals were dissolved in 100% DMSO. The optical density was measured at a wavelength of 570 nm and a background wavelength of 620 nm using a multi-functional microplate reader Spark 10M (Tecan, Männedorf, Switzerland). The cytotoxicity index (IC_50_) was determined from dose–response curves using the median effect analysis according to the method [64].

**Cellular accumulation of nanoparticles.** Cells were plated on coverslips in 6-well culture plates at 3 × 10^5^ cells per well. Next, 24 h after sieving, the test compounds were added to the incubation medium: DOX and DOX–copolymer composition at a concentration of 10 µmol∙L^−1^ according to DOX, and VP-TEGDM copolymer at a concentration of 1 mg∙mL^−1^. Six hours after applying the compounds, the cells were washed repeatedly with PBS, and were fixed with 4% formaldehyde. Then, permeabilization was carried out in a 0.2% Triton X-100 solution (Panreac, Castellar del Vallès, Spain), and the nuclei were stained with a fluorescent dye 4,6-diamidino-2-phenylindole dihydrochloride (DAPI, Serva, Heidelberg, Germany). Microscopy was performed using a fluorescence microscope Zeiss Axio Scope.A1 (Zeiss, Jena, Germany) and filters Fs49 (excitation G365 nm, emission BP445/50 nm, Zeiss A-Plan objective, 40×/0.65) and Fs45 (excitation BP560/40 nm, emission BP630/75 nm, Zeiss A-Plan lens, 40×/0.65). Image visualization was performed using an AxioCam MRc5 camera (Zeiss, Jena, Germany) and Zen software (Zen 2 lite, Zeiss).

## 3. Results and Discussion

### 3.1. VP-TEGDM and VP-MAA-TEGDM Copolymer Parameters and Properties

**Figure 2:**
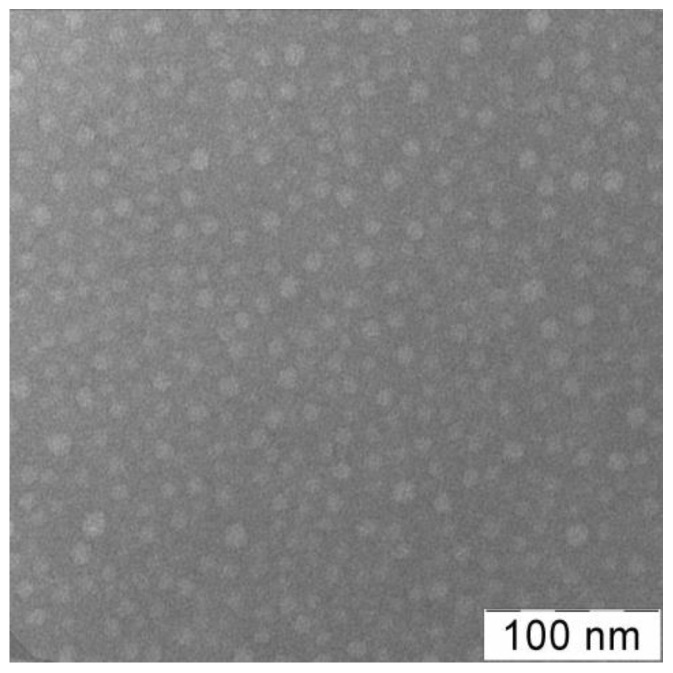
TEM image of VP-TEGDM copolymer [53]. It was obtained from the aqueous solution (0.1 mg mL^−1^) using Leo 912 AB equipment (Leo Electron, Oberkochen, Germany). Phosphotungstic acid was used to contrast the sample.

The VP-TEGDM and VP-MAA-TEGDM copolymers (Figure 1b,c) have been obtained by radical copolymerization in toluene and ethyl alcohol, respectively. The bifunctional monomer TEGDM, like MMA and MAA [65,66], was more reactive in radical copolymerization than VP, according to our kinetics study of copolymerization in a wide range of VPs and dimethacrylate conversions and analysis of the monomer composition of the resulting copolymers by IR spectroscopy and isothermal calorimetry [67]. The time dependences of conversion of dimethacrylate and VP monomers calculated from the decrease in the intensity of corresponding absorption bands in IR spectra showed that the rate of VP conversion was much lower than that of dimethacrylate. As a result, at the initial stage of copolymerization, all radicals added more active monomers, and polymer chains formed were enriched with MAA and TEGDM unites. Their distributions in growing polymer chains were statistical and stimulated the limitation of intermolecular cross-linking leading to insoluble macrogel formation. It can be assumed that the polymer structure formed at the initial stages of the reaction contained “pendant” C=C bonds of TEGDM units, through which growing PVP chains were attached. This is supported by the two-step pattern of the dependence of the reduced rate of VP copolymerization with dimethacrylate on conversion in ethanol. The first portion of the kinetic curve located in the region of initial and average conversions (up to ~0.3) was attributed to the radical copolymerization of comonomers. The second portion was related to the grafting polymerization of VP onto the copolymer containing pendant C=C bonds; therefore, the content of VP units in the copolymer increased. Reactivity ratios of VP (*M*_1_) and MAA (*M*_2_) were *r*_1_ = 0.04 and *r*_2_ = 0.56 [66], and MAA (*M*_1_) and MMA (*M*_2_), the monofunctional analog of TEGDM, *r*_1_ = 0.32 and *r*_2_ = 0.60 [68]; hence, MAA was more active as TEGDM in radical copolymerization than VP. As a result, 3D copolymer structures (Figure S1) consisted of branched fragments enriched with MAA and TEGDM units and long PVP chains. They can be considered as micelle-like formations in which there were polar and low-polarity moieties. An analysis of a cryo-TEM image [69] of the fullerene C60 hybrid structure based on the VP-TEGDM copolymer obtained in the presence of 1-decanethiol indicated its micellar nature. The fullerene particles of about 3 nm size were visible in the core of micellar particles with diameter ca. 25–30 nm. The typical TEM image of the VP-TEGDM copolymer is presented on Figure 2. The micrograph shows spherical particles with a size of 10–12 nm.

The VP units prevailed in the polymer chains as follows from the average molar composition of the copolymers, calculated from the elemental analysis data (Table 1). The total content of (di)methacrylate units was higher in the terpolymer. According to potentiometric titration, it contained ca. 4 wt% of MAA units, had close to the VP-TEGDM copolymer absolute weight average molecular weight *M*_w_ and was characterized by a sufficiently narrow polydispersity coefficient PD compared to the copolymers synthesized by radical copolymerization. Commonly, they have a wide molecular mass distribution as a result of the statistical nature of radical processes. For example, the copolymer of MAA with divinylbenzene prepared at a molar ratio of 100:2:1 in the presence of dodecanethiol as a chain transfer agent was characterized by PD ~ 55 [70]. To control the molecular mass and PD of the VP copolymer, we also used similar regulators [67]. However, their application led to the inclusion of the residues of such molecules into polymer chains, and the pollution of the copolymer by unreacted compounds. The copolymer synthesis is complicated, and its cost is rising; therefore, special cleaning methods are required. The branched nature of the VP-MAA-TEGDM copolymer was demonstrated in [52] through an analysis of the dependence of *M*_w_ on the eluent volume *V*_r_ and root-mean-square radii of gyration on *M*_w_ values and comparison with those for linear PVP.

**Figure 3:**
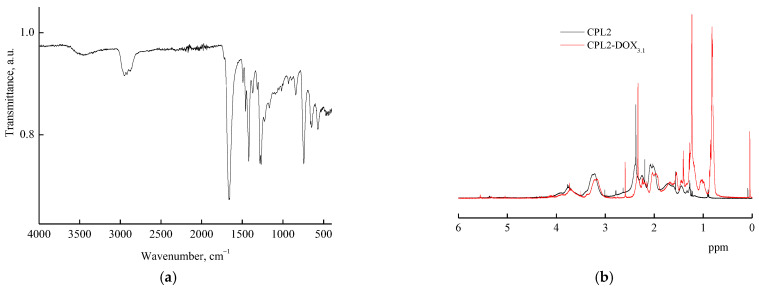
FTIR spectrum (**a**) and ^1^H NMR spectra (**b**) of VP-MAA-TEGDM copolymer (CPL2) and DOX–copolymer composition (CPL2-DOX_3.1_).

The VP-TEGDM copolymer was characterized by IR spectroscopy and NMR spectroscopy [53]. In the VP-MAA-TEGDM spectrum, absorption bands at wavenumbers ~1720 and ~1665 cm^−1^, respectively, corresponding to the stretching vibrations of C=O groups in (di)methacrylate and VP units were observed (Figure 3a). There was a broad absorption band typical of the stretching vibrations assigned to OH groups of adsorbed water linked by hydrogen bonds to amphiphilic copolymer [43] in the range of 3600–3000 cm^–1^. The water absorption hindered the identification of MAA units from the corresponding absorption bands in this spectral range.

The ^1^H NMR spectrum of the methacrylic acid, proton of the OH group was registered at δ = 11 ppm; however, in the spectra of the copolymers, this signal was absent (Figure 3b), apparently due to a low content of these units in polymer chains or an insufficient concentration of copolymers in solutions. At the same time, signals typical of protons of VP and TEGDM units were observed. For example, the ^1^H NMR spectra showed two groups of signals corresponding to VP units. The first group included signals at δ = 3.0–4.0 ppm due to NCHα protons of polymer chains and CH_2_C=O moieties of pyrrolidone. The second group was composed of signals at δ = 1.4–2.4 ppm related to protons of CH_2_ moieties in polymer chains and C–CH_2_–C and NCH_2_ moieties of pyrrolidone. Signals assigned to protons of CH_3_ groups in TEGDM units were observed in the spectrum of the copolymers at δ ~ 0.9 ppm. The ^1^H NMR spectrum exhibited a broad signal at δ = 4.1 ppm related to hydrogen atoms in a –CH_2_–CH_2_– moiety of the reacted TEGDM. However, at δ ~ 5.6 and 6.2 ppm, there were weak signals corresponding to protons in pendant C=C bonds of TEGDM.

**Figure 4:**
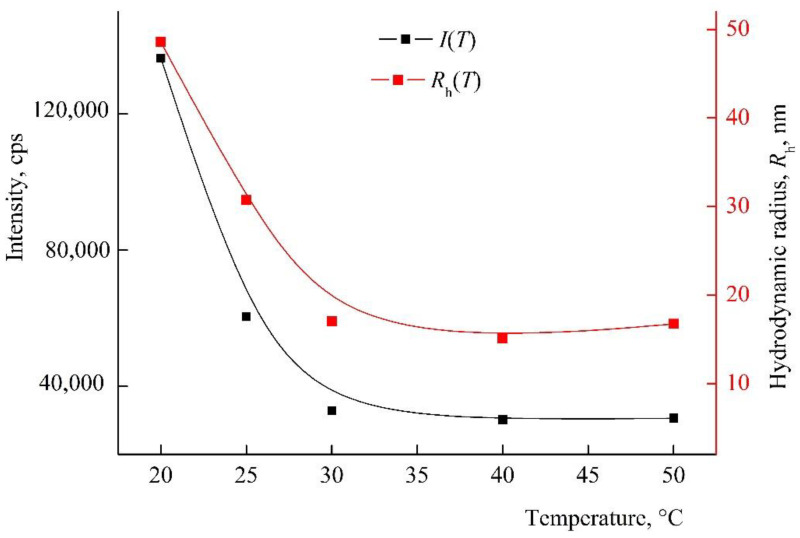
The dependencies of the light scattering intensity *I* and the hydrodynamic radius *R*_h_ of light scattering centers on temperature by VP-TEGDM copolymer alcohol solution. The copolymer concentration in IPA is 7 mg mL^−1^. Red symbols refer to the right *y*-axis.

The amphiphilic VP-TEGDM copolymer dissolved in both media: polar as water, alcohol, DMSO, *N*-methylpyrrolidone and low-polar as chloroform, toluene, etc. The behavior of the amphiphilic VP-TEGDM copolymer was investigated at differed concentrations in IPA and PBS by the DLS method. It is seen from Figure 4 that the average value of the intensity of light scattering *I* and the hydrodynamic radius *R*_h_ at the maximum of the main peak decreased with the temperature (the distribution of particles on sizes is given in Figure S2). The average value of *I* decreased by more than 3 times with the temperature increase from 20 to 30 °C, and then it did not change, as with the *R*_h_ value, too. In the temperature range of 30–50 °C, stable scattering centers of less than 20 nm size were present in the solution. This could be due to an increase in the solubility of the copolymer in IPA, and the decomposition of aggregates into small structural units that were stable in this temperature range.

**Figure 5:**
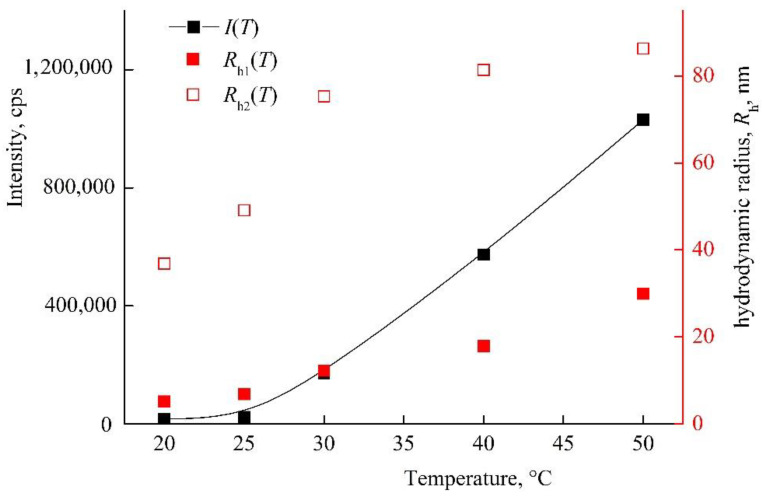
Dependencies of light scattering intensity *I* and hydrodynamic radius *R*_h_ of light scattering centers in PBS on temperature at VP-TEGDM copolymer concentration of 1.2 mg mL^−1^. Red symbols refer to the right *y*-axis.

Figure 5 shows *I*(*T*) and *R*_h_(*T*) dependencies of the VP-TEGDM copolymer in PBS. It should be stressed that solutions with a higher concentration were noticeably opalescent at room temperature. Note that the size distribution of the intensity of scattering centers was bimodal, and the solution contained two types of particles with *R*_h1_ and *R*_h2_ (Figure S3). As the temperature rose, their *R*_h_ values increased and reached ~30 and ~90 nm at 50 °C. *I*(*T*) and *R*_h_(*T*) dependencies allowed concluding that the VP-TEGDM copolymer was thermo-responsive. Some opalescence of the copolymer solution was observed near the lower critical solution temperature as a result of aggregation of the copolymer scattering centers. The copolymer ability to temperature respond was maintained in slightly acidic (pH 5) and neutral (pH 6.8–7.4) media, in contrast to the VP-MAA-TEGDM terpolymer.

**Figure 6:**
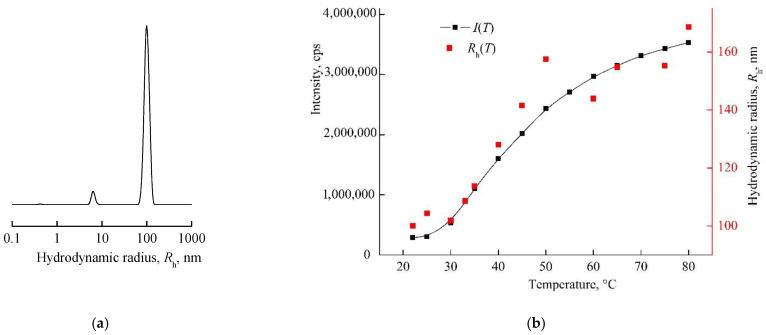
The light scattering distribution on dimensions of scattering centers in the aqueous solution of the 5 mg mL^−1^ VP-MAA-TEGDM copolymer (**a**) and dependencies of light scattering intensity *I* and hydrodynamic radius *R*_h_ of the solution on temperature (**b**). Red symbols refer to the right *y*-axis.

The intensity of light scattering by neutral/weakly alkaline and alkaline aqueous solutions of VP-MAA-TEGDM in the studied temperature interval remained almost invariable, and polymer particles with the hydrodynamic radius smaller than 10 nm were presenting in these solutions [52]. Here, we showed DLS data for the nanosized DOX system based on this terpolymer in water, i.e., slightly acidic medium. The size distribution was close to unimodal in an aqueous solution of the VP-MAA-TEGDM copolymer (5 mg mL^−1^), and the peak on a distribution curve respected the aggregates of 100 nm size (Figure 6a). The intensity of light scattering of the aqueous solution of this copolymer started to increase noticeably at *T* > 30 °C, and *R*_h_ values of scattering centers were growing until ~150 nm (Figure 6b), i.e., its aggregation was getting stronger, and the solution became visibly opalescent. These changes, as in the case of the VP-TEGDM copolymer, were reversible: when the solution had cooled, it again became transparent, and the particle sizes decreased as a result of aggregates’ destruction. Previously, we have shown [43] that water molecules bond with the oxygen C=O of VP lactam ring, ether and carbonyl groups of TEGDM units. The addition enthalpy (−∆*H*) of one water molecule on the C=O groups of VP units for the VP-VP-VP copolymer moiety was 4–6 kcal mol^−1^ and 2–4 kcal mol^−1^ on the ether and carbonyl groups of the TEGDM unit [43]. The H-bonds between (di)methacrylates units and water were primarily destroyed with increasing temperature, and the solubility of the copolymer decreased. Thus, the 3D structures of VP-copolymers reacted on a temperature change in aqueous solutions. They can be loaded with BAC, and their controlled release from a carrier can occur via the temperature effect [39].

### 3.2. Experimental Study of DOX–Copolymer Structures’ Formation in Alcohol or Aqueous Solutions and Their Physical and Chemical Properties

Using the VP-TEGDM copolymer as an example, the process of DOX encapsulation into polymer particles in alcoholic or aqueous solutions was studied in detail. According to DLS, IPA was a more thermodynamically suitable solvent for an amphiphilic copolymer than water. The dimensions of polymer aggregates were lower in the alcohol than in water at a copolymer concentration of 7 mg mL^−1^, and the temperature range of 20–25 °C. Moreover, hydrophilic DOX was poorly soluble in IPA that facilitated its association with the copolymer. Drug association with the copolymer can be through physical entrapment of the drug within the polymer matrix, penetration of the DOX molecules into their cavities with formation of the complexes such as the “guest-host” type and drug adsorption on the surface of nanoparticles.

In aqueous copolymer solutions at 20 °C (Figure S3), there were two types of scattering centers with sizes of ca. 5 and 36 nm that can be loaded with DOX. Upon dilution of the solution, the distribution of scattering centers became broad, which can lead to a wide size distribution of DOX–copolymer systems. Moreover, DOX molecules can remain in water in a free form in an aqueous solution of a copolymer because water is a good solvent for it, and some of the macromolecules can remain non-loaded. In this regard, the choice of solvent for the encapsulation of DOX in the VP-TEGDM copolymer was an important factor in the design of nanoscale systems. The organic solvent was likely to promote the association of DOX and copolymer to a greater extent than water. The process of encapsulation of the guest molecule inside the cavity of the macromolecular system will be determined by dispersion forces, electrostatic interactions and hydrogen bonds. Thus, the encapsulation of DOX into dendrimers based on phenylene, thiophene, etc., was regulated by both π-π-stacking and σ-type interactions, with some contribution of hydrogen bonds [33].

#### 3.2.1. The Study of Aqueous Solutions of DOX Polymer Structures by Electronic Absorption Spectroscopy

Intermolecular interactions in aqueous solutions of DOX polymer structures were studied using electron adsorption spectroscopy as one of the simplest and most efficient methods of analysis. It has been previously shown that in the concentration range 10^−5^–10^−4^ M DOX in water, the Bouguer–Lambert–Beer law is fulfilled, i.e., DOX in water is in molecular form or small aggregates [71], and there are no intermolecular interactions with the solvent.

**Figure 7:**
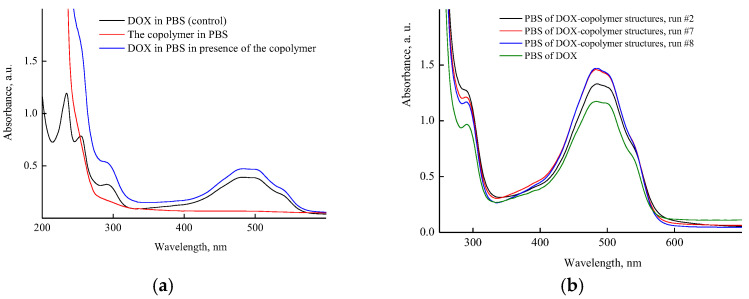
Absorption spectra: (**a**) PBS of VP-TEGDM copolymer, DOX in the control experiment (4.3 × 10^−5^ M) and a copolymer nanostructure with 0.4 wt% DOX (run #1); (**b**) PBS of DOX in control experiment (1.3 × 10^−4^ M) and DOX–copolymer structures (runs #2, 7, 8). All descriptions of experiments are given in Table S1.

Figure 7a shows the absorption spectra of aqueous buffer solutions of DOX, the copolymer, and the DOX–copolymer nanostructure (run #1, Table S1) prepared by the 1st manner. It can be seen that in the region above 275 nm, the copolymer solution was optically transparent, and its spectrum overlapped with the DOX absorption bands in the UV region. The DOX absorption spectrum showed absorption bands with maxima at wavelengths of 255, and 292 nm, as well as it presented a broad absorption band of a complex shape in the visible region with the maximum at a wavelength of ~490 nm. In the presence of the copolymer, the optical density of the DOX band in a visible region changed insufficiently (Figure 7a). However, as is seen from Figure 7b, the optical density of the DOX absorbance band depended on the copolymer content in a solution. It increased with respect to the control experiment, apparently as a result of the polymer’s surrounding influence on its absorbance. The hyperchromic effect increased at small amounts of the copolymer in a solution (0.4 and 1.5 mg mL^−1^), and DOX content per the copolymer was 15 and 5 wt%, respectively. Thus, the copolymer concentration in solution was an important factor for DOX nanostructures’ preparation.

**Figure 8:**
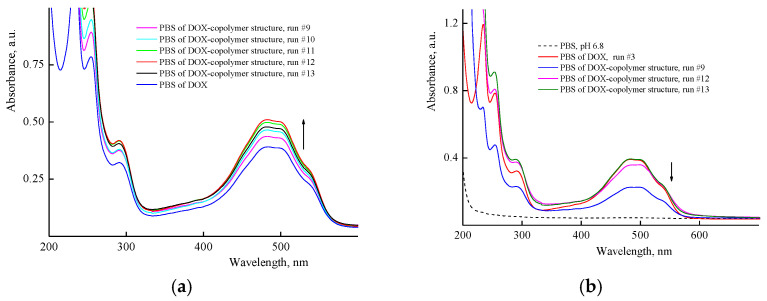
Absorption spectra of aqueous solutions of DOX in the presence of various copolymer concentrations recorded immediately after encapsulation by second manner, and DOX solution (4.3 × 10^−5^ M) in the control experiment, run 3 (**a**) and PBS of DOX nanostructures obtained in 9, 12 and 13 runs (**b**).

Figure 8a shows the UV-vis spectra of aqueous solutions of DOX in the presence of different concentrations of the copolymer just after the encapsulation by the manner two, i.e., with using the copolymer aqueous solutions. It can be seen that the optical density of the band at ~490 nm increased (hyperchromic effect) with the concentration of the copolymer in the solution to reach a limiting value at 5% of DOX per copolymer, and then decreased, apparently as a self-aggregation result of DOX molecules in polymer particles or interaction with functional groups of the copolymer. Moreover, the absorption spectra of DOX were changed upon the dissolution of DOX composition in PBS (Figure 8b). The optical density of DOX absorption bands decreased by a factor of ~2 in solutions of polymer structures with the high DOX content (40%) obtained in a very diluted copolymer solution (0.0625 mg mL^−1^), i.e., the hypochromic effect was observed. The optical density values of an absorbance band of the polymer structure with low DOX content (2.5%) and free DOX were practically the same. This means that in these polymer particles, DOX was either in the native state and did not interact with the polymer or was released from the polymer particles into the solution. Thus, the optical characteristics of DOX–copolymer structures in solutions depend on the copolymer concentration used at their formation, DOX content per the copolymer and medium properties.

#### 3.2.2. Behavior of DOX–Copolymer Nanostructures in an Aqueous Neutral Buffer Solution

**Figure 9:**
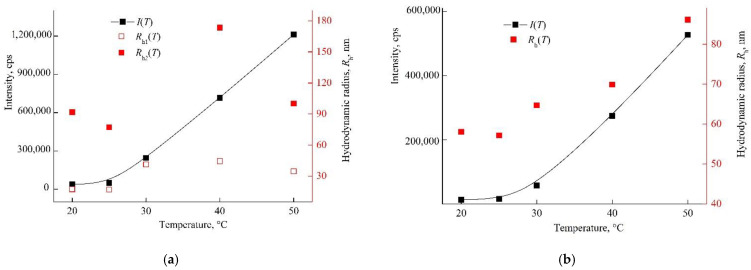
Dependencies of the light scattering intensity by PBS of DOX–copolymer structures and the hydrodynamic radius of scattering centers on temperature at 1.2 (**a**) and 0.4 (**b**) mg mL^−1^ DOX–copolymer composition, respectively (Table S1, runs #1 and 2). Red symbols refer to the right *y*-axis.

The behavior of DOX polymer structures obtained by the 1st manner (runs #1 and #2, Table S1) and contained 0.4 and 1.1 wt% of the drug per the copolymer was studied by DLS in PBS. For this, the initial solutions of DOX nanostructures (runs #1 and #2, Table S1) were diluted, respectively, up to concentrations of 1.2 and 0.42 mg mL^−1^ relative to the copolymer. As the temperature rose, the intensity of light scattering sharply increased in both the solutions (Figure 9), and they became opalescent at 40 °C due to the aggregation of scattering centers. The size distribution of these centers was polymodal and depended on the temperature and the concentration of DOX polymer structures in the solution (Figure S4). With the diluted solution, the distribution became narrower, and the *R*_h_ value at the maximum of the main peak decreased, which indicated the aggregate nature of the scattering centers. It follows from DLS that solutions of the DOX–copolymer structures were thermally sensitive, too, as with the initial copolymer. Thus, DOX delivering from the polymer particles can be controlled via temperature.

Solutions of DOX in PBS (10^−4^–10^−5^ M) were unstable, and the drug precipitated over time. Within 2 weeks, the optical density of the absorption band of DOX solutions in the visible region decreased almost twice (Figure S5a). At the same time, the DOX structure based on the terpolymer was much more stable, and the decrease in the optical density of its solution did not exceed ~20% (Figure S5b). Thus, the inclusion of DOX in polymer particles increases its stability in PBS and prevents the drug precipitation due to aggregation.

#### 3.2.3. Cyclic Voltammetry of the Free DOX and Being Encapsulated in VP-MAA-TEGDM Copolymer

Molecular electrochemistry now is closely interfaced with medicinal chemistry, especially in the case of quinones [72]. Voltammetry allows obtaining additional information about the properties of nanosystems as encapsulated into (co)polymer electrochemical active compounds [44,45,53,57,73], especially if their free (molecular) and encapsulated forms differ substantially with energetic characteristics, see, e.g., [45,52,57].

Being electroactive, too, DOX was two-electron and practically reversible reduced on liquid and solid electrodes (Hg and amalgams, Au, Pt, C, etc.) in protogenic media on the quinone reaction center, and oxidized quasi-reversible or practically irreversible and also two-electron on the hydroquinone moiety (positions 5,12 and 6,11, respectively, Figure 1a). Numerous data were obtained at first on Hg [74,75,76,77,78,79] and later on solid electrodes, and foremost on carbonic ones [56,77,78,79,80,81] (GC [56], pyrographite, carbon paste electrode, etc. [77,78,79,80,81,82]).

**Figure 10:**
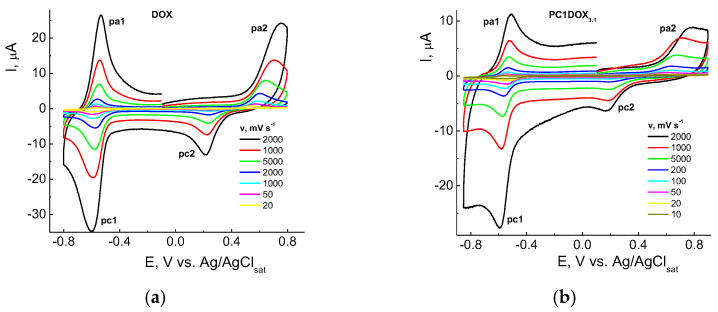
CVA curves of DOX (**a**) and CPL2-DOX_3.1_ composition (**b**) in PBS (pH 7.24) on a GC-electrode at the scan rates *v* = 10–2000 mV s^−1^. DOX concentrations are 4.0 × 10^−5^ (**a**) and 1.4 × 10^−5^ M (**b**).

In general, the mechanism of DOX electrode reactions was analogous to classic quinone derivatives [83]. CVA curves were similar within the potential *E* range ca. from −1 to +1 V on solid electrodes, and on Hg from 0 to −1 V. They contained cathodic-anodic peaks on the cathodic branch at the potentials ca. −(0.45–0.65) V, and anodic-cathodic ones near +(0.3–0.7) V on the anodic branch depending on the pH, electrode nature and other experimental conditions. Similar CVA curves were also recorded in our study for different DOX concentrations, and those encapsulated into the copolymer, namely, CPL2-DOX_3.1_ composite in PBS on a GC-electrode within a broad range of potential scan rate *v* = 10–2000 mV s^−1^ (Figure 10).

**Figure 11:**
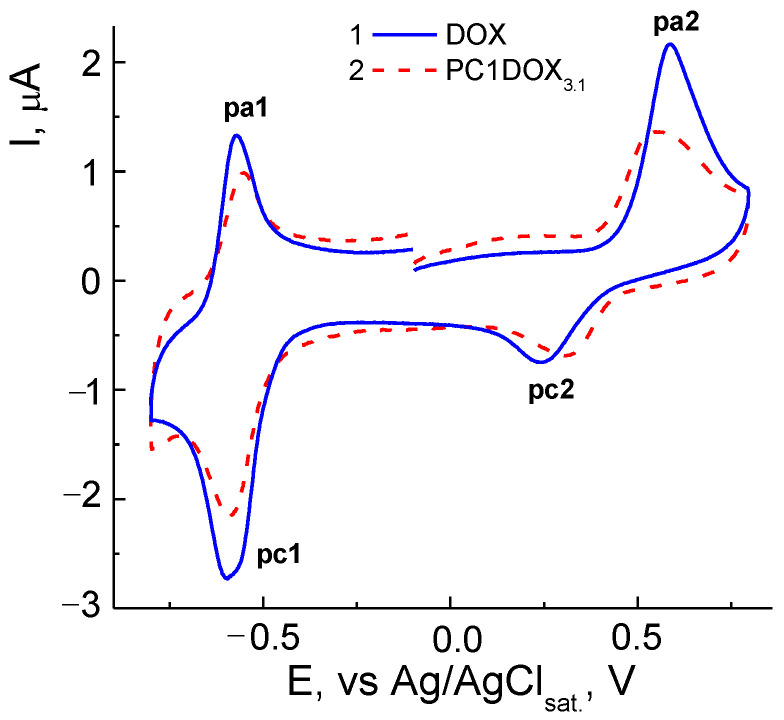
CVA curves of DOX and CPL2-DOX_3.1_ in PBS in coordinates *I*, *E* at *v* = 100 mV s^−1^. DOX concentrations are 4.0 × 10^−5^ (*1*) and 1.4 × 10^−5^ M (*2*).

Two couples of the redox peaks on CVA curves in the negative (pc1/pa1) and positive (pa2/pc2) potential regions were associated with the reduction in quinone (pc1) and oxidation of hydroquinone centers (pa2) in the DOX molecule according, e.g., to [76,77]. The system of peaks in the cathodic area near −0.6 V vs. Ag/AgCl respected the nearly reversible process, and oxidation within the +(0.3–0.6) V range was practically irreversible. However, there were some differences in the electrochemical behavior of DOX (Figure 10a) and CPL2-DOX_3.1_ (Figure 10b). For a vivid comparison, curves recorded at *v* = 100 mV s^−1^ from Figure 10 were represented for these forms (Figure 11). Therefore, the reduction (pc1) of DOX in CPL2-DOX_3.1_ occurred somewhat more difficult, and oxidation (pa1) was somewhat easier with respect to the non-bonded form of DOX that led to more reversibility of the process. The situation was somewhat different in the anodic region: for CPL2-DOX_3.1_, the pc2 peak was shifted to more positive potentials, whereas the pa2 peak was shifted to less positive *E*. Consequently, encapsulation retarded the reduction/oxidation of the quinone moiety of DOX and facilitated the oxidation/reduction of the hydroquinone moiety. In other words, the oxidation of the encapsulated form occurred more easily, and reduction was more difficult, although the potential difference did not exceed some tenth of the mV. The reason can be in the participation of these groups in the formation of a hydrogen bond, and its stretching and weakening. Moreover, the nature of the electrode process itself was changed: it transmitted from purely adsorption for non-bonded DOX to a mixed adsorption–diffusion process for DOX in CPL2-DOX_3.1_. It followed from the slopes of the dependences from Figure 11 replotted in lg *I*, lg *v* coordinates that consisted of 0.92 and 0.74, respectively.

The interactions between DOX and the terpolymer were also manifested in the ^1^H NMR spectra. The ^1^H NMR spectra of CPL2-DOX_3.1_ in deuterated chloroform (Figure 2b) showed the displacement of the signals related to VP units of the terpolymer to lesser values of the magnetic field, probably as a result of the intermolecular interactions with DOX. According to NMR [84], the H-bonds and/or dipole–charge interactions determined the interaction in supramolecular structures of daunorubicin, a related DOX drug, and polymer nanoparticles.

### 3.3. Theoretical Investigation of Polymer Structures of DOX in Solutions

#### 3.3.1. Quantum-Chemical Modeling of the Structure of DOX–Copolymer Complexes

**Figure 12:**
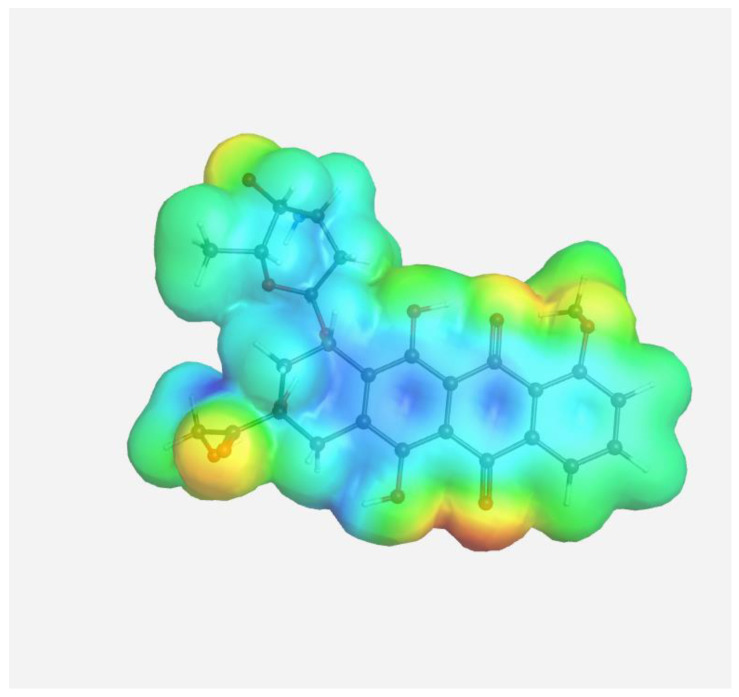
Electrostatic potential of the DOX molecule; red regions correspond to negative electrostatic potentials, and blue regions correspond to positive electrostatic potentials.

The experimental data on intermolecular interactions in the DOX–copolymer system were confirmed by the results of quantum-chemical modeling. As shown in [85], in DOX structures based on VP copolymers, the hydrogen bonds were the main intermolecular bonds. Figure 12 shows the surface of the electrostatic potential for the DOX molecule, containing numerous functional groups and hydrogen atoms able to form hydrogen bonds.

It can be seen that the largest negative charge was concentrated on the oxygen atoms of the C=O groups, followed by methoxy and OH groups. They most likely will take part, as electron donors, in the formation of intermolecular bonds. The largest positive charge was at the hydrogen atoms of the OH and NH_2_ groups; therefore, they are also expected to form bonds between the copolymer and the guest molecule. From the same point of view, a section of the copolymer was considered, consisting of VP and TEGDM units in a molar ratio corresponding to the experimental molar composition, and a cavity of the branched VP-TEGDM copolymer was simulated.

**Figure 13:**
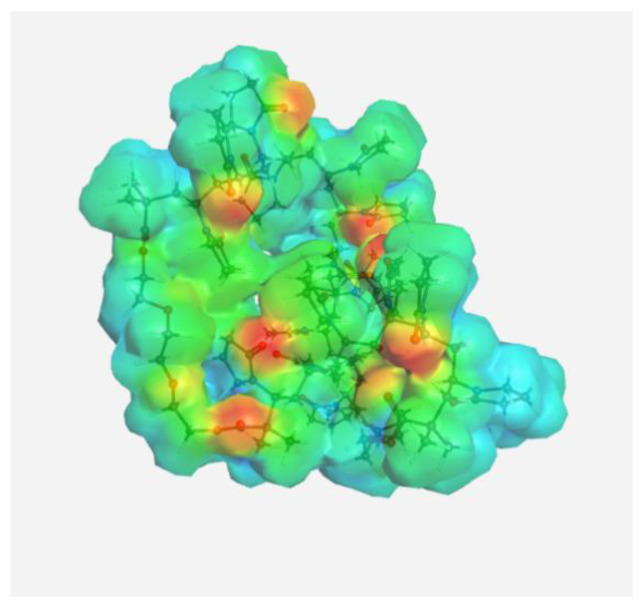
Electrostatic potential of the VP-TEGDM copolymer cavity.

**Figure 14:**
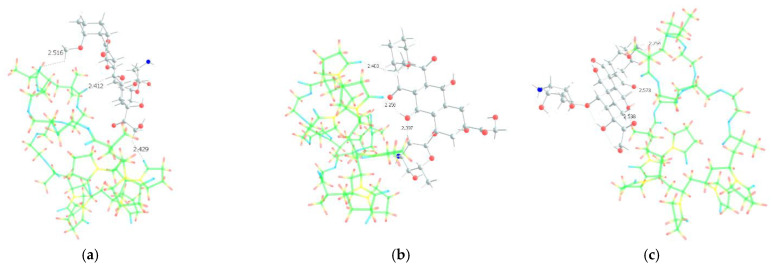
Optimized geometries of DOX–copolymer complex obtained from AM1 (Gaussian) and PBE/SBK (PRIRODA). The lengths of intermolecular hydrogen bonds are given. (**a**) structure 1, (**b**) structure 2, (**c**) structure 3.

As we assumed earlier, the most negative were the oxygen atoms C=O of the VP lactam ring, as well as the C=O group of the TEGDM units. The negative charge on the oxygen of the ether group was weaker and, in fact, the containing TEGDM unit moieties were a positive charge (Figure 13). Based on this, several structures of the complex were modeled (Figure 14).

It can be seen that one DOX molecule formed several intermolecular bonds with the copolymer site at once, which was not surprising, given the large number of variants in their formation. As expected, the main electron donor in this system was the C=O group of the lactam ring, and the DOX hydrogen atoms as the acceptors. However, there were also bonds formed by oxygen atoms of DOX and hydrogen atoms of the copolymer, especially if the drug molecule was coordinated near the TEGDM units, although these bonds turned out to be weaker (Figure 14).

In order to study the characteristics of the formed bonds, their modeling was carried out. For this, using DFT, we optimized a short region (3 units) of the copolymer with a DOX molecule bound by a specific hydrogen bond. Further, the obtained geometries were studied using the QTAIM method. The parameters of hydrogen bond in the structures of DOX–copolymer units are given in Table 2.

As we assumed earlier [85], the studied bonds were hydrogen ones in all parameters; however, they differed greatly in strength. The bond energies of the carbonyl group of the VP lactam cycle and the hydrogen atoms of the OH and NH_2_ groups as an electron donor and acceptors were close to typical values. At the same time, the bonds formed with the participation of the oxygen atom of the ether group of the TEGDM unit were extremely weak.

#### 3.3.2. Modeling of TDDFT Absorbance Spectra of DOX and Its Complexes

**Figure 15:**
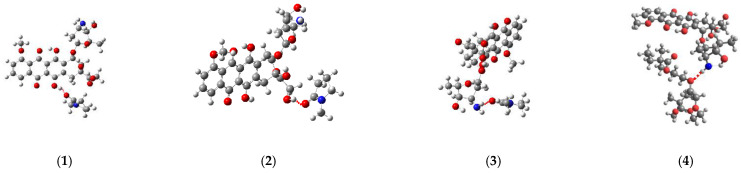
Structures of the DOX complexes with VP (**1**–**3**) and TEGDM (**4**) copolymer units.

The simulation of the TDDFT spectra was a more computationally demanding task. Therefore, to solve it, the interaction of DOX with one unit of the VP-TEGDM copolymer was considered. This should not introduce a serious error, since the characteristics of the intermolecular bonds considered by us should not depend on the length of the polymer chain. Several complexes with the hydrogen bonds were modeled to simulate the TDDFT spectra (Figure 15).

**Figure 16:**
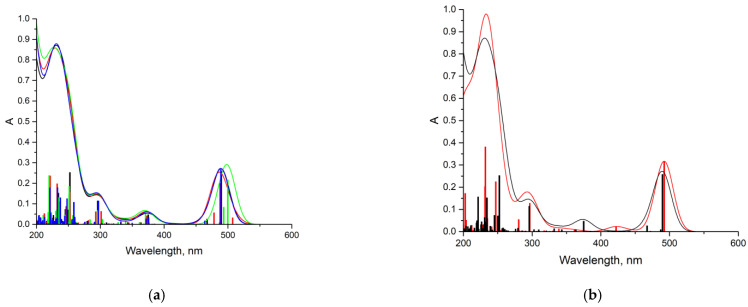
TDDFT spectra: (**a**) DOX (black) and DOX-VP unit complexes (green—complex **1**, red—complexes **2**, blue—complex **3**); (**b**) DOX (black) and DOX-TEGDM unit complex **4** (red); thin lines in the graph correspond to electronic transitions, and wide bands in the graph are obtained by applying Gaussian broadening to the lines (*k* = 30.85).

**Figure 17:**
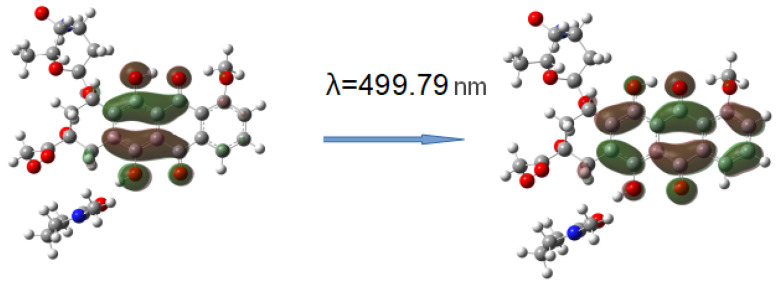
HOMO-LUMO electron transition in DOX-VP unit complex **1**.

Figure 16 shows the TDDFT spectra of these complexes; for comparison, the TDDFT spectrum of the optimized geometry of the DOX molecule satisfactorily coincided with the experimental one. If in complexes **1**–**3** the oxygen atom C=O of the VP group acted as an electron donor during the formation of a hydrogen bond, then the spectrum practically coincided with the DOX spectrum. An exception was complex 1: a shift of the characteristic DOX band in the visible region by 10 nm was observed in its spectrum, accompanied by some increase in its intensity. This is because this band was responsible for the π→π* HOMO-LUMO electronic transition in the π-system of conjugated rings in both DOX and complex **1** (Figure 17). The bathochromic shift of this band was most likely due to the interaction of the molecular orbitals of the VP with the π-system of conjugated DOX rings, which insignificantly decreased the energy of this transition from 2.53 to 2.48 eV, according to the calculations.

Consequently, the shift and increase in the intensity of the band in the visible region can be associated with the formation of this hydrogen bond due to the C=O group of the VP and OH group of DOX, which was not surprising, since due to the distribution of the electrostatic potential in DOX and in the copolymer, they were the most likely, and according to the QTAIM analysis, the most durable. In the theoretical spectra, this situation was observed also in complex **4** (H-bond formation due to ester oxygen atoms of TEGDM). Consequently, despite the low energy of bonds with TEGDM units, their formation also took place in the system, possibly even as an addition to the main bonds due to VP units, which were also associated with steric reasons. Thus, the calculations show that DOX molecules were bound to the functional groups of the copolymer, which were electron donors, due to hydrogen bonds of different strength and energetics. It allowed controlling the release of the drug from the copolymer by breaking these bonds in the physiologically important temperature range.

### 3.4. Biological Activity of Encapsulated DOX in VP-TEGDM Copolymer

**Figure 18:**
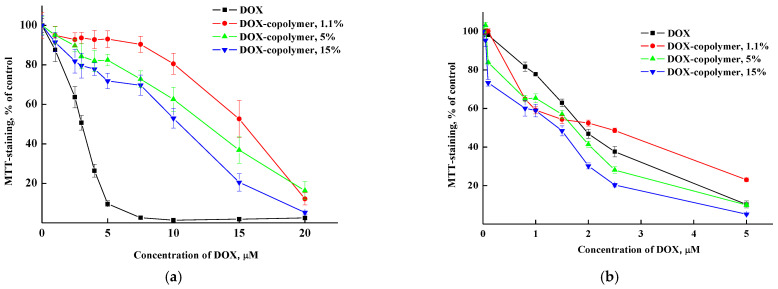
Dose–effect curves for *HeLa* cells treated with DOX and copolymer composition containing 1.1, 5 and 15 wt% DOX for 24 (**a**) and 72 (**b**) h exposures.

To study the biological activity of encapsulated DOX, dry copolymer-based compositions obtained in the runs #2, 7 and 8 were used (Table S1). The DOX content was about 1.1, 5 and 15% per the copolymer. The cytotoxic study was carried out using an MTT test on *HeLa* tumor cell culture. We have previously shown [42] that the copolymer has low toxicity to cells and, thus, will not contribute significantly to the increase in the encapsulated DOX toxic effect. In the experiments, we used two exposures with compounds: 24 and 72 h. The data are shown in Figure 18.

The dose IC_50_ values’ calculation showed that after 24 h of action, all copolymer compositions with DOX had significantly lower cytotoxicity compared to free DOX (Table 3). At the same time, after 72 h, the differences in the toxic effect on cells between DOX and its encapsulated form were minimal.

One of the possible explanations for the differences in the cytotoxicity of free and encapsulated DOX after 24 h is the delayed release of DOX distributed in different parts of copolymer particles and their intermolecular interactions, including hydrogen bonds. The more DOX is contained in polymer particles, the more it accumulates in cells and the cytotoxic effect is higher. The absence of differences in the cytotoxicity of free and encapsulated DOX after 72 h is the most likely, due to its complete release from the copolymer.

**Figure 19:**
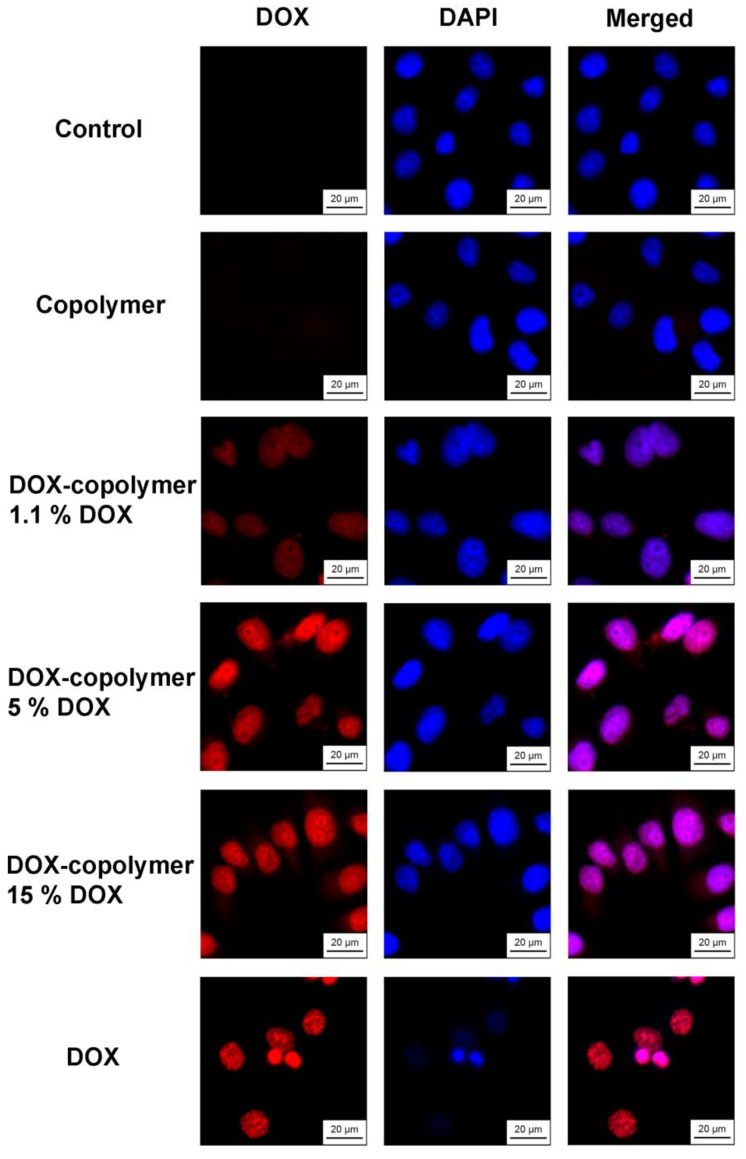
Intracellular accumulation of DOX and copolymer compositions containing 1.1, 5 and 15 wt% DOX after 6 h of exposure to *HeLa* cells. The concentration of DOX is 10 μmol L^−1^; the cell nuclei are stained with DAPI dye. Magnification: ×40.

Fluorescence microscopy was used to study differences in the intracellular accumulation of free and encapsulated forms of DOX. Solutions of DOX–polymer compositions were added at the same concentration of 10 μM (in terms of DOX), and a comparison was made of the DOX accumulation after 6 h of exposure to cells. DAPI dye was used to visualize cell nuclei. The results are shown in Figure 19.

It was shown that after 6 h of action, DOX accumulated in the cell nuclei and caused a change in their size, which was a consequence of its toxic effect. Under the same conditions, polymer compositions containing 5 and 15 wt% of DOX were actively accumulated in the cell nuclei, which was evident from the fluorescence of DOX. However, no visual changes in cell morphology were observed. A significantly lower fluorescence of DOX was characteristic of the polymer composition containing 1.1 wt% of DOX. The obtained microscopic data corresponded to the differences in the cytotoxicity of the studied compounds for *HeLa* cells. The accumulation of DOX in cells when using the polymer compositions containing 5 and 15 wt% of DOX, apparently, was insufficient for the manifestation of a cytotoxic effect, to the same extent as with the action of free DOX.

It is also important that when using a polymer composition containing 5 or 15 wt% DOX, its fluorescence was observed in the cell cytosol, while free DOX completely passed into the nuclei during the experiment. Thus, after 6 h of action on cells, a part of DOX was released from the polymer and interacted with nuclear DNA, while a part remained in the particles and was released later, which was confirmed by cytotoxicity data for 72 h.

## 4. Conclusions

Nanosized DOX systems of high biological activity in vitro were obtained by the drug encapsulating into the biocompatible thermo-responsive copolymers VP-TEGDM and VP-MAA-TEGDM, and they were characterized by a set of physical chemical methods. The results of quantum-chemical modeling indicate the formation of hydrogen bonds of different strengths and energies between oxygen atoms of various functional groups of VP, TEGDM units and OH, NH_2_-groups of DOX, and make it possible to predict the structure of nanosized DOX systems and the possibility to control their behavior under the influence of the temperature for the controlled release of the active substance. DOX will be retained in 3D copolymer structures due to the intermolecular interaction, in particular H-bonds’ formation, and will be released with the increasing temperature as a result of their destruction and structural transformation of the polymer carrier. The results of biological studies show that the low-toxic VP copolymers can serve as a basis to develop the targeted delivery vehicles for DOX. Thus, the encapsulation of DOX into 3D copolymer structures makes it possible to diminish the toxic effect in vitro due to its slower accumulation in cells. The introduction of a vector molecule into the developed DOX systems for “recognition” of only tumor cells will allow the creation of a promising tool for the targeted delivery of the antitumor antibiotic.

## Figures and Tables

**Table 1:** Physicochemical characteristics of the VP-TEGDM and VP-MAA-TEGDM copolymers.

The Copolymers	The Content of VP and (Di)methacrylates Units in Copolymers, wt%	Size Exclusion Chromatography (RI + MALLS)	
		*M*_w_, kDa	*PD*
VP-TEGDM	90.6:9.4	62.3	3.4
VP-MAA-TEGDM	85.6:14.4	76.0	2.1

**Table 2:** Hydrogen bond parameters from QTAIM method for optimized (tpssh/6-31G*) sites.

Structures	The Bond Length, Å	ρ, a.e.	∇^2^ ρ(r), a.e.	*E*_bond_, kcal mol^−1^
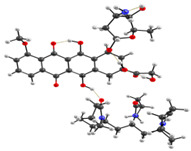	1.790	0.044	0.139	11.8
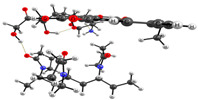	1.807	0.035	0.112	9.4
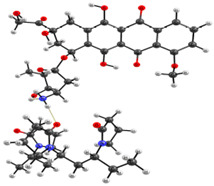	1.968	0.026	0.079	6.8
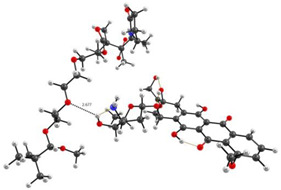	2.677	0.005	0.024	1.2
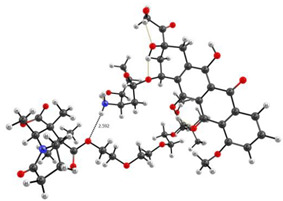	2.592	0.006	0.025	1.3

**Table 3:** Dose IC_50_ values of DOX and DOX–copolymer compositions for *HeLa* cells.

Compound/Copolymer Composition	DOX Content per the Copolymer, wt%	Dose IC_50_, μM ^1^	
		24 h	72 h
DOX	100	3.23 ± 0.39	1.58 ± 0.19
DOX-copolymer	1.1	13.90 ± 2.56 *	2.45 ± 0.23 *
	5.0	11.18 ± 2.25 *	1.69 ± 0.10
	15.0	8.83 ± 2.37 *	1.37 ± 0.09

^1^ Statistical significance was assessed using a *t*-test. The criterion of statistical significance was * *p* < 0.05.

## Data Availability

Not applicable.

## Supplementary Materials

The following supporting information can be downloaded at: https://www.mdpi.com/article/10.3390/pharmaceutics14122572/s1, Table S1: Concentrations of reagents and conditions for DOX encapsulation in the VP-TEGDM copolymer (CPL1) according to the manner 1; Table S2: Concentrations of reagents and conditions of DOX encapsulation into the VP-TEGDM copolymer according to the manner 2. Description of structure and properties of copolymers; Figure S1: Topological structures of VP-TEGDM (a) and VP-MAA-TEGDM (b) copolymers; Figure S2: The intensity distribution of light scattering over the size of scattering centers by a solution of the VP-TEGDM copolymer in IPA (7 mg mL^−1^) on temperature; Figure S3: The intensity distribution of light scattering over the size of scattering centers by an aqueous buffer solution of the VP-TEGDM copolymer (1.2 mg mL^−1^) on temperature. Description of an aqueous buffer solution of the DOX–copolymer; Figure S4: The intensity distribution of light scattering over the size of scattering centers by an aqueous buffer solution of the DOX–copolymer obtained in run 1 (a) and run 2 (b) on temperature. The concentrations are 1.2 and 0.42 mg mL^−1^, resp.; Figure S5: Absorption spectra of DOX (a) and DOX–terpolymer structure (b) in PBS (initial and over two weeks). Cuvette is 1 cm.
