# Supplementary material for: New Nanosized Systems Doxorubicin—Amphiphilic Copolymers of N-Vinylpyrrolidone and (Di)methacrylates with Antitumor Activity
Source: Pharmaceutics. 2022 Nov 23;14(12):2572. doi: 10.3390/pharmaceutics14122572 (PMC9784683; doi:10.3390/pharmaceutics14122572)
Supplement: Supplementary file 1 [file pharmaceutics-14-02572-s001.zip › pharmaceutics-1972181-supplementary.pdf]

pharmaceutics

New Nanosized Systems Doxorubicin—Amphiphilic Copolymers of *N*-Vinylpyrrolidone and (Di)methacrylates with Antitumor Activity

Svetlana V. Kurmaz [1,*], Vladislav M. Ignatiev [1], Nina S. Emel'yanova [1], Vladimir A. Kurmaz [1], Dmitry V. Konev [1], Anastasiya A. Balakina [1] and Alexey A. Terentyev [1,2,3]

[1] Federal Research Center of Problems of Chemical Physics and Medicinal Chemistry, Russian Academy of Sciences, ac. Semenov av. 1, 142432 Chernogolovka, Russia
[2] Faculty of Fundamental Physical and Chemical Engineering, Moscow State University M.V. Lomonosov, GSP-1, Leninskie Gory, 1, Building 51, 1 Educational Building, 119991 Moscow, Russia
[3] Scientific and Educational Center of the Moscow State Regional University, St. Vera Voloshina 24, 141014 Mytishchi, Russia
* Correspondence: skurmaz@icp.ac.ru; Tel.: +7-496-522-10-89

Description of conditions for DOX encapsulation in the VP-TEGDM copolymer; Table S1: Concentrations of reagents and conditions for DOX encapsulation in the VP-TEGDM copolymer (CPL1) according to the manner 1, Table S2: Concentrations of reagents and conditions of DOX encapsulation into the VP-TEGDM copolymer according to the manner 2. Description of structure and properties of copolymers; Figure S1: Topological structures of VP−TEGDM (a) and VP−MAA−TEGDM (b) copolymers, Figure S2: The intensity distribution of light scattering over the size of scattering centers by a solution of the VP-TEGDM copolymer in IPA (7 mg mL^{-1}) on temperature, Figure S3: The intensity distribution of light scattering over the size of scattering centers by an aqueous buffer solution of the VP-TEGDM copolymer (1.2 mg mL^{-1}) on temperature. Description of an aqueous buffer solution of the DOX-copolymer, Figure S4: The intensity distribution of light scattering over the size of scattering centers by an aqueous buffer solution of DOX-the copolymer obtained in run 1 (a) (and run 2 (b) on temperature. The concentrations are 1.2 and 0.42 mg mL^{-1}, resp., Figure S5: Absorption spectra of DOX (a) and DOX-terpolymer structure (b) in PBS (initial and over two weeks). Cuvette is 1 cm.

Citation: Kurmaz, S.V.; Ignatiev, V.M.; Emel'yanova, N.S.; Kurmaz, V.A.; Konev, D.V.; Balakina, A.A.; Terentyev, A.A. New Nanosized Systems Doxorubicin—Amphiphilic Copolymers of *N*-Vinylpyrrolidone and (di)Methacrylates with Antitumor Activity. *Pharmaceutics* **2022**, *14*, 2572. https://doi.org/10.3390/pharmaceutics14122572

Received: 29 September 2022
Accepted: 18 November 2022
Published: 23 November 2022

Publisher's Note: MDPI stays neutral with regard to jurisdictional claims in published maps and institutional affiliations.

Copyright: © 2022 by the authors. Submitted for possible open access publication under the terms and conditions of the Creative Commons Attribution (CC BY) license (https://creativecommons.org/licenses/by/4.0/).

Table S1. Concentrations of reagents and conditions for DOX encapsulation in the VP-TEGDM copolymer (CPL1) according to the manner 1.

# runs	Volume ratio of the CPL1 solutions in IPA and DOX in water, ml	$[CPL1]_0$, mg ml^{-1}	$[DOX]_0$, mg ml^{-1}	DOX content per CPL1, %	pH 0.01 M neutral water buffer solution
1	4:0.1	7.0	1.0	0.4	7.0
2	4:0.3	7.0	1.0	1.1	7.0
3	4:0.1	0.0	1.0	–	7.0
4	4:0.0	7.0	0.0	–	7.0
5	4:0.3	7.0	0.0	0.0	7.0
6	4:0.1	7.0	1.0	0.4	6.8
7	4:0.3	1.5	1.0	5.0	6.8
8	4:0.3	0.5	1.0	15.0	6.8

Table S2. Concentrations of reagents and conditions for DOX encapsulation in the VP-TEGDM copolymer (CPL1) according to the manner 2.

# runs	Volume ratio of the CPL1 solutions in IPA and DOX in water, ml	$[CPL1]_0$, mg ml^{-1}	$[DOX]_0$, mg ml^{-1}	DOX content per CPL1, %	pH 0.01 M neutral water buffer solution
9	4:0.1	0.0625	1.0	40	7.0
10	4:0.1	0.1250	1.0	20	7.0
11	4:0.1	0.2500	1.0	10	7.0
12	4:0.1	0.5000	1.0	5	7.0
13	4:0.1	1.000	1.0	2.5	7.0

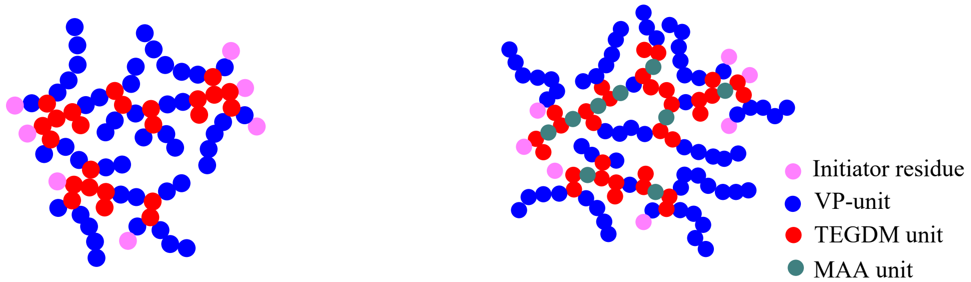

(**a**) (**b**)

Figure S1. Topological structures of VP–TEGDM (**a**) and VP–MAA–TEGDM (**b**) copolymers.

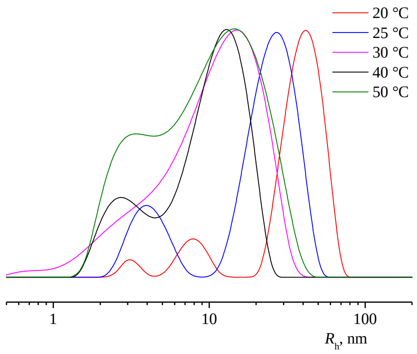

Figure S2. The intensity distribution of light scattering over the size of scattering centers by a solution of the VP-TEGDM copolymer in IPA (7 mg mL^{-1}) at different temperatures.

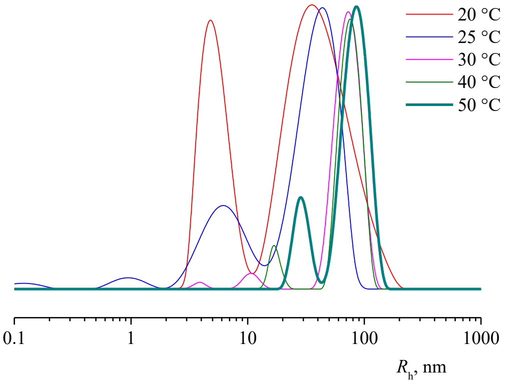

Figure S3. The intensity distribution of light scattering over the size of scattering centers by PBS (pH 7.0) of the VP-TEGDM copolymer (1.2 mg mL^{-1}) at different temperatures.

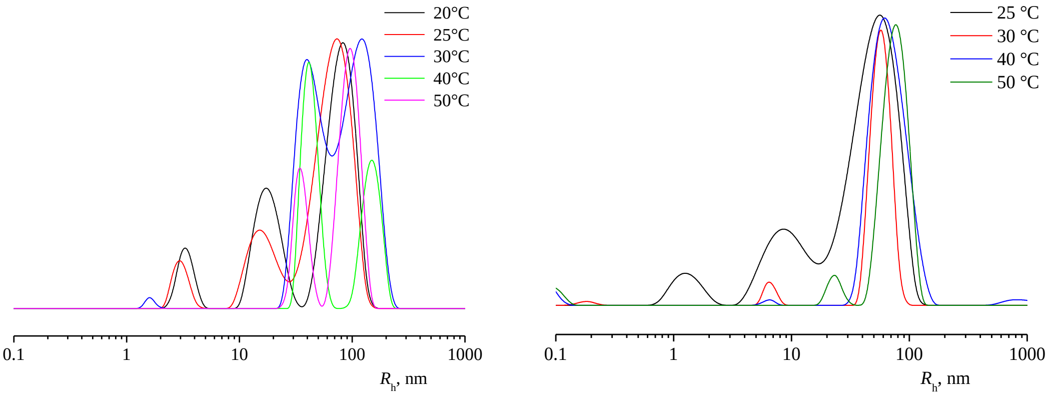

(**a**) (**b**)

Figure S4. The intensity distribution of light scattering over the size of scattering centers by PBS (pH 7.0) of DOX-copolymer compositions obtained in run 1 (**a**) and run 2 (**b**) at different temperatures. The concentrations are 1.2 and 0.42 mg mL^{-1}, resp.

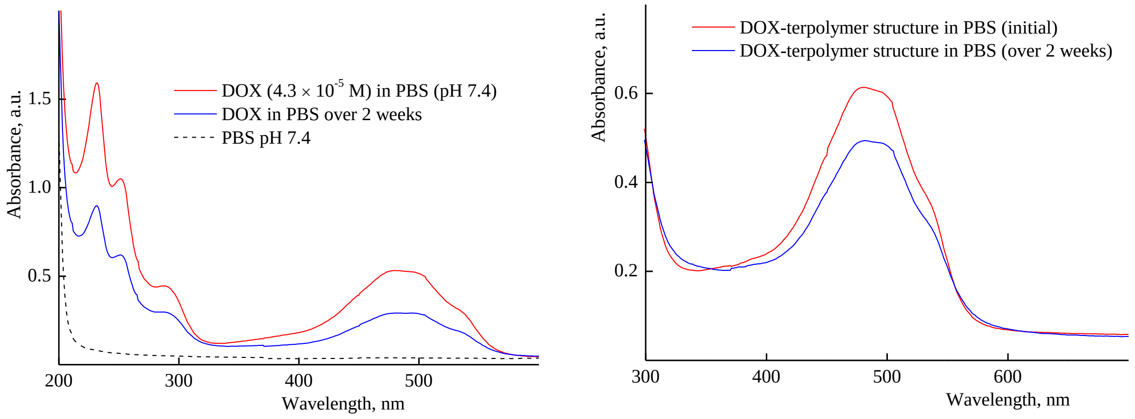

(**a**) (**b**)

Figure S5. Absorption spectra of DOX (**a**) and DOX-terpolymer structure (**b**) in PBS (initial and over two weeks). Cuvette is 1 cm.
